# Supplementary material for: YB-1 Knockdown Inhibits the Proliferation of Mesothelioma Cells through Multiple Mechanisms
Source: Cancers (Basel). 2020 Aug 14;12(8):2285. doi: 10.3390/cancers12082285 (PMC7464182; doi:10.3390/cancers12082285)
Supplement: Supplementary file 1 [file cancers-12-02285-s001.zip › cancers-885241-XML supply.docx]

Article

YB-1 Knockdown Inhibits the Proliferation of Mesothelioma Cells through Multiple Mechanisms

Thomas G. Johnson, Karin Schelch, Kaitao Lai, Kamila A. Marzec, Marina Kennerson, Michael Grusch, Glen Reid and Andrew Burgess


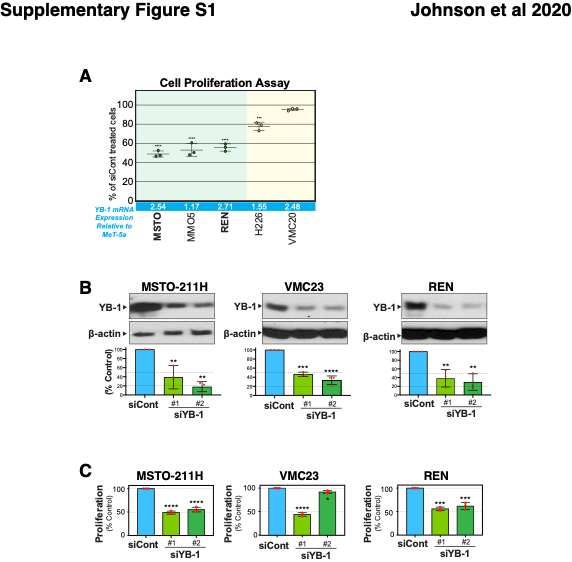


**Figure S1.** Validation of YB-1 siRNA target sequences. (**A**) SYBR-based Cell Proliferation assay of MPM cell lines treated with our without (siCont) siYB-1#1 for 96 h. Green bar indicates MPM cell lines that responded well to YB-1 siRNA (<60% inhibition compared to siCont), yellow bar indicates MPM cell lines that only responded weakly or showed no significant change. The relative fold change in YB-1 mRNA expression compared to MeT-5A, an immortalised non-malignant mesothelial cell line, as previously published [12]. (**B**) Immunoblots of whole protein harvested from MPM cells after transfection with either control (siCont) or one of two YB-1 siRNA (siYB-1 #1 and #2) (Figure S8). The primary YB-1 antibody was the polyclonal rabbit anti-YB-1 ab12148 (Abcam). Monoclonal mouse β-actin antibody (Sigma Aldrich) served as the loading control. (**C**) SYBR Green based proliferation assays of MSTO-211H, VMC23 or REN cells transfected with siCont or siYB-1 (#1 or #2). All data is presented as a percentage of siCont transfected cells. ***p* < 0.01, ****p* < 0.001, *****p* < 0.0001.

**
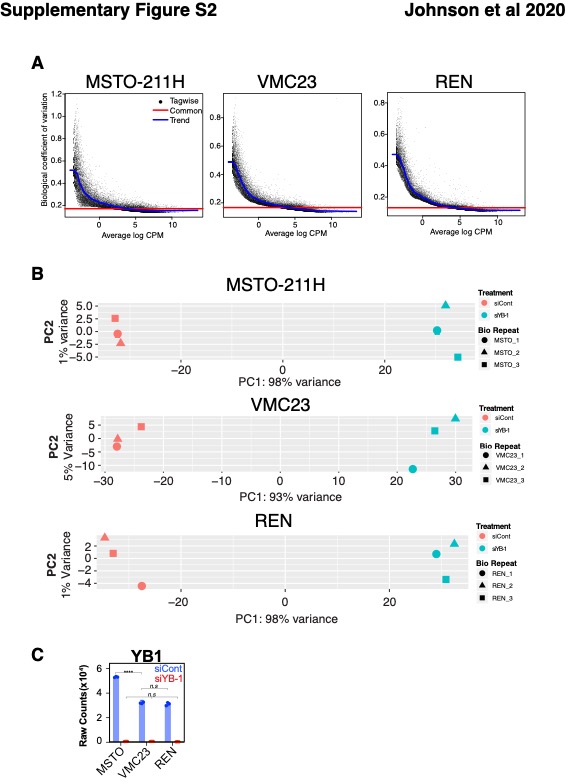
 Figure S2.** RNA-Seq data validation. (**A**) Biological coefficient of variation plots for RNA-seq datasets depicting number of differentially expressed genes. (**B**) Principal components analysis plots showing the variance due to siRNA treatment and replicate variability. Both sets of plots were generated using R programming software. (**C**) Raw counts of YBX1 mRNA expression in each MPM cell line after transfection with siCont or siYB-1. Not significant (n.s), **** *p* < 0.0001.


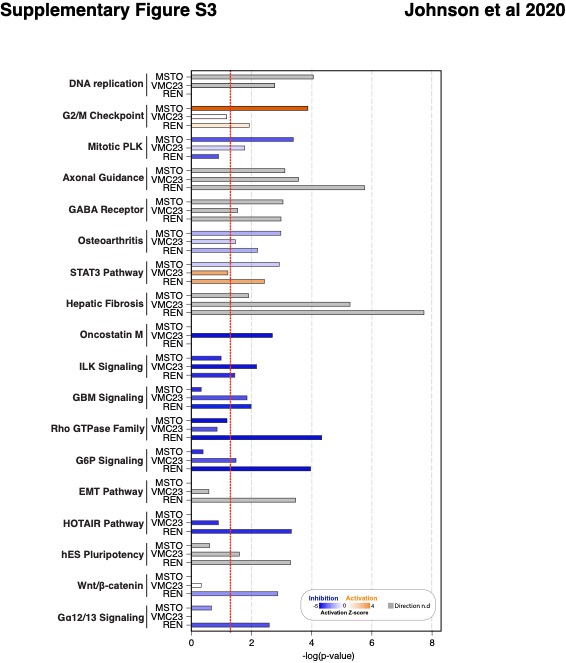


**Figure S3.** Altered pathways across cell lines. IPA canonical pathway analysis of the RNA-seq data. Predictions of inhibition (blue) or activation (orange) or no change (white) states are based on the Ingenuity® Knowledge Base, which compares the expected change with experimental observation to all known upstream regulators. Red line indicates significance (*p* < 0.05). Grey bars indicate where no directionality could be determined (Direction n.d).

**Figure S4.** Uncropped Western Blot figures of Figure 4E.


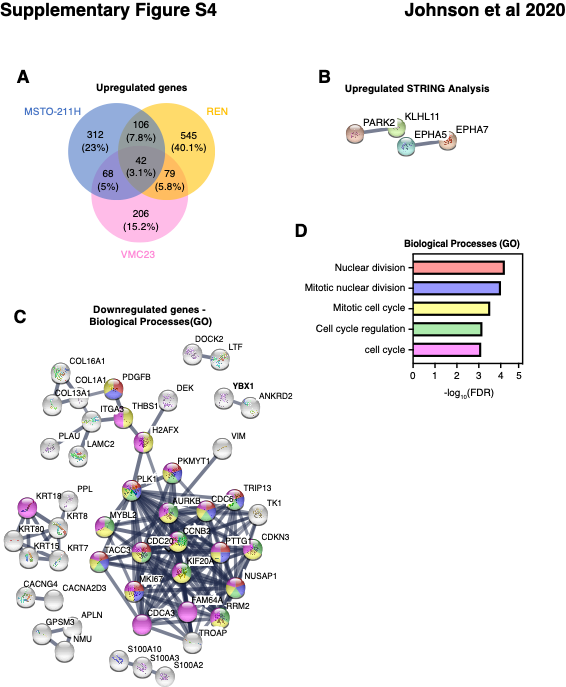


**Figure S5.** Common genes altered across all three cell lines. (**A**) Venn diagram of all significantly upregulated genes (log2(fold change) < -2; *q*-value (FDR) < 0.001) in each cell line, displayed as number of genes (percent of total genes across three cell lines). (**B**) STRING-DB Reactome Pathway analysis of common upregulated human genes (n = 42) across all three cell lines. Network edges are displayed as ‘confidence’ and disconnected nodes are hidden. (**C**) STRING-DB Biological Processes (GO) analysis of common downregulated human genes (n = 150) across all three cell lines. Network edges are displayed as ‘confidence’ and disconnected nodes are hidden. Genes highlighted in colour correspond to (**D**) the top five most significant Biological Processes (GO) pathways. Significance is shown as -log10(FDR).


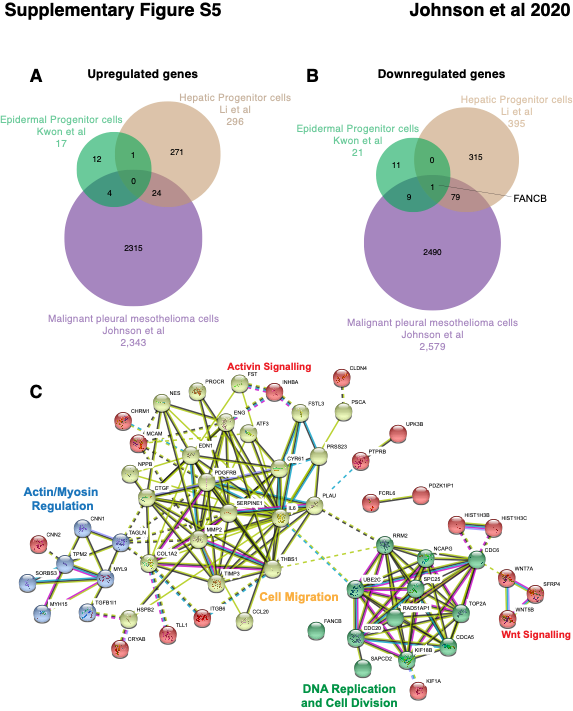


**Figure S6.** Comparison with previous studies. Venn diagrams of all significantly (**A**) upregulated and (**B**) downregulated genes across the Kwon et al, Li et al and the present RNA-seq datasets. (**C**) STRING-DB analysis of all common downregulated genes between any of the three studies (n = 1 (Kwon/Li/Johnson) + 9 (Kwon/Johnson) + 79 (Li/Johnson) = 89). Colours represent enriched pathways for clusters. Network edges are displayed as ‘evidence’ and disconnected nodes are hidden.


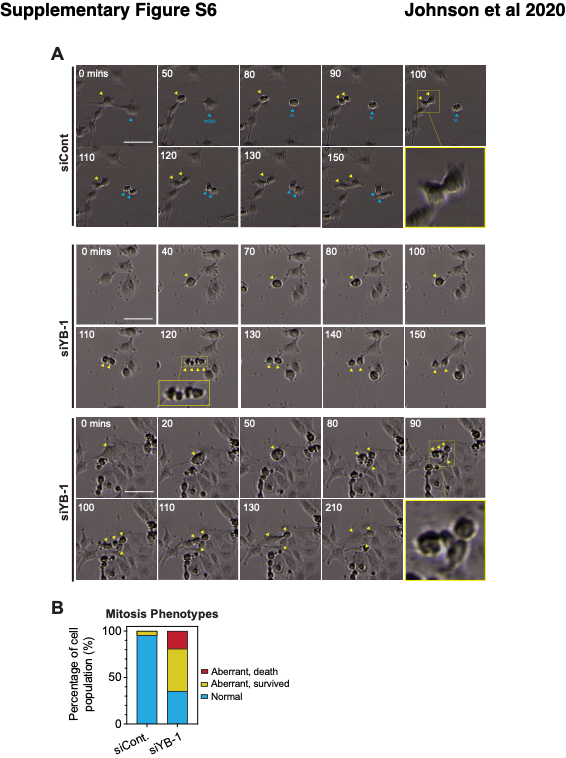


**Figure S7.** YB-1 cytokinesis defects. (**A**) Videomicroscopy photographs of REN cells transfected with either siCont or siYB-1 #1 undergoing mitosis. Yellow and blue arrows indicate cells of interest. Time (min), scale bars = 50 μm. (**B**) Number of all mitotic events counted in Figure 7 and 8 for REN cells in each treatment group that were normal (blue), aberrant resulting in survival (yellow) and aberrant resulting in cell death (red). Data is the percentage of all mitotic events in the respective treatment group.


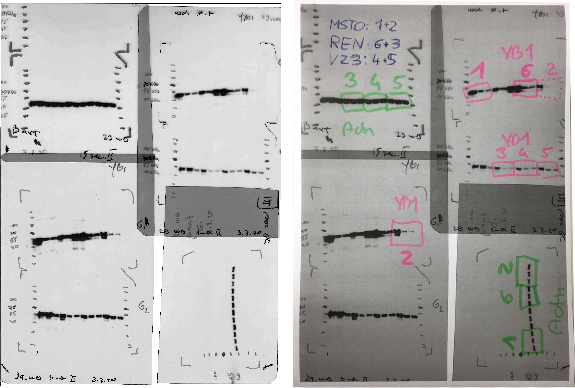


**Figure S8.** Uncropped Western Blot figures of Figure S1B.

.

| 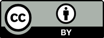 | © 2020 by the authors. Licensee MDPI, Basel, Switzerland. This article is an open access article distributed under the terms and conditions of the Creative Commons Attribution (CC BY) license (http://creativecommons.org/licenses/by/4.0/). |
| --- | --- |
